# Photocatalytic Depolymerization of Native Lignin toward Chemically Recyclable Polymer Networks

**DOI:** 10.1021/acscentsci.2c01257

**Published:** 2022-12-28

**Authors:** Hongyan Wang, Gavin J. Giardino, Rong Chen, Cangjie Yang, Jia Niu, Dunwei Wang

**Affiliations:** Department of Chemistry, Merkert Chemistry Center, Boston College, Chestnut Hill, Massachusetts 02467, United States

## Abstract

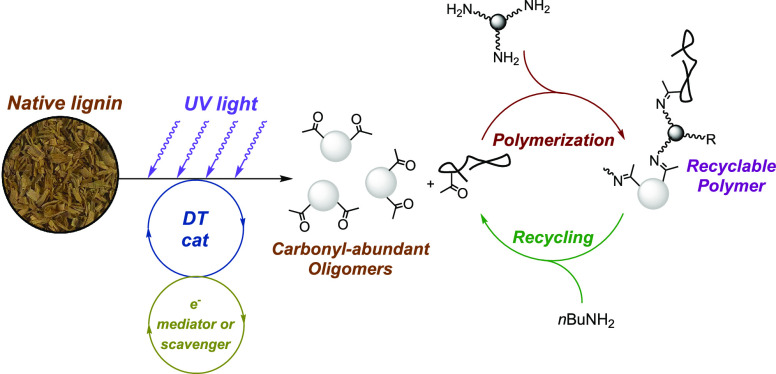

As an inedible component of biomass, lignin features rich functional groups that are desired for chemical syntheses. How to effectively depolymerize lignin without compromising the more valuable cellulose and hemicellulose has been a significant challenge. Existing biomass processing procedures either induce extensive condensation in lignin that greatly hinders its chemical utilization or focus on fully depolymerizing lignin to produce monomers that are difficult to separate for subsequent chemical synthesis. Here, we report a new approach to selective partial depolymerization, which produces oligomers that can be readily converted to chemically recyclable polymer networks. The process takes advantage of the high selectivity of photocatalytic activation of the β-O-4 bond in lignin by tetrabutylammonium decatungstate (TBADT). The availability of exogenous electron mediators or scavengers promotes cleavage or oxidation of this bond, respectively, enabling high degrees of control over the depolymerization and the density of a key functional group, C=O, in the products. The resulting oligomers can then be readily utilized for the synthesis of polymer networks through reactions between C=O and branched −NH_2_ as a dynamic covalent cross-linker. Importantly, the resulting polymer network can be recycled to enable a circular economy of materials directly derived from biomass.

## Abstract 2

The photocatalytic depolymerization of native lignin is explored for generating carbonyl-abundant oligomers that can undergo cross-linking to form recyclable dynamic polymer networks.

## Introduction

As an important component of lignocellulose, lignin offers numerous advantages as an attractive feedstock. For example, lignin is abundant, accounting for 15–40% of the total biomass;^1^ it is rich in aromatic functionalities that are of great potential value for chemical synthesis and material fabrication; lignin is inedible, so its utilization will not compete with food needs.^2,3^ However, existing biomass processing technologies prioritize cellulose and hemicellulose.^4^ As a result, lignin has been significantly underutilized.^5^ Consider the traditional pulping process as an example. The delignification methods produce the so-called technical lignin, which often leads to structural heterogeneity and undesired side reactions (e.g., condensation) and makes its subsequent chemical utilization challenging.^5,6^ Recently, an alternative lignin-first strategy has emerged to directly convert native lignin in lignocellulose into value-added chemicals.^4,7,8^ For instance, reductive catalytic fractionation (RCF) as a lignin-first approach produces a mixture of low molecular weight compounds from native lignin.^9−13^ However, the mixture produced by RCF is often difficult to separate. Moreover, RCF tends to destroy high-value functional groups such as carboxylic acids, aldehydes, and aromatic rings, undermining the value of these products as precursors for chemical syntheses.^9,14−16^ Indeed, most RCF studies focus on retrieving the thermal energy of the products by using them as fuels.

**Figure 1:**
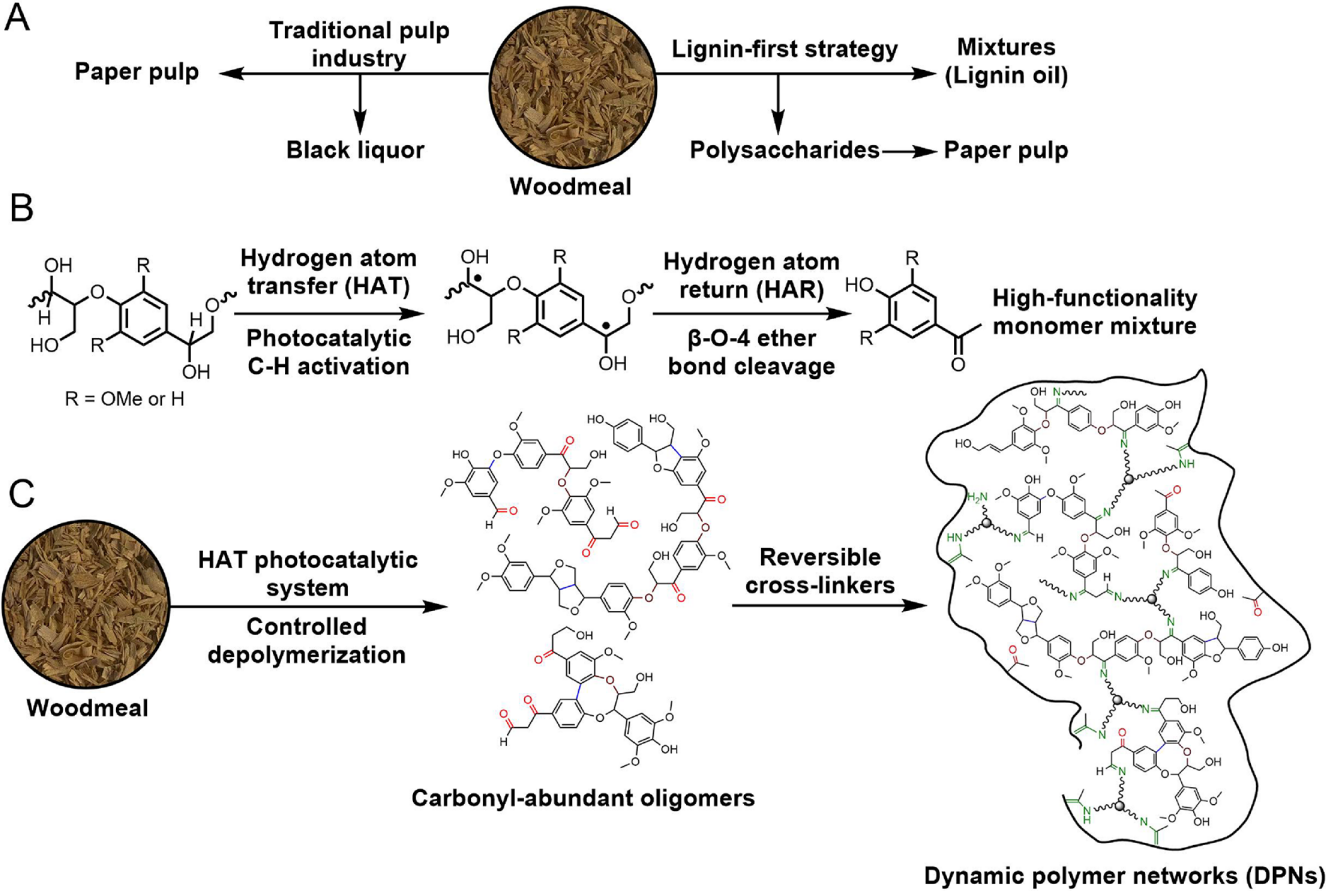
Schematic illustration of different strategies to process lignocellulose. (a) Two industrial routes of processing woodmeal, namely, the pulp industry or the lignin oil industry. (b) Mechanisms of hydrogen-atom transfer to break the β-O-4 motif, which is abundant in native lignin. (c) Overview of our strategy to first depolymerize native lignin and then repolymerize the resulting oligomers using dynamic covalent cross-linkers.

Recognizing these challenges, researchers have recently turned their attention to depolymerizing native lignin under mild conditions. Successful examples have been demonstrated to utilize the hydrogen-atom transfer (HAT) reaction for selectively targeting the abundant β-1 and β-O-4 motifs.^17−25^ A unique advantage offered by HAT is the ability to preserve the aromatics, ketones, and aldehydes.^17,21^ Nevertheless, earlier attempts of using HAT-based chemistries for lignin valorization have mostly focused on producing small molecules, which remain challenging to separate.^18,22^ On the other hand, partial depolymerization of lignin has started to show its promise for the construction of functional materials, such as thermoset plastics,^26−29^ elastomers,^30^ or vitrimers.^31^ Nevertheless, these initial materials are constructed from kraft lignin,^31^ which has already undergone significant unwanted chemical modifications in the pulping process that affects its chemical integrity.^5,6^ We see from this discussion the significance of a lignin-first approach, in which native lignin is selectively depolymerized for subsequent repolymerization. To preserve the high-value cellulose and hemicellulose, the depolymerization should take place under mild conditions. Here, we report a proof of this concept (Figure 1). Our work is inspired by previous reports on the cleavage of the β-O-4 motif in lignin via HAT.^18−23,25^ To achieve the intricate balance between depolymerization and the introduction of functional groups necessary for the subsequent repolymerization, we employ an earth-abundant photocatalyst (namely, tetrabutylammonium decatungstate, or TBADT)^32^ that enables HAT under mild conditions. The reaction can be guided through either a bond-scission or an oxidation pathway through exogenous electron mediators or electron scavengers, respectively, thereby regulating the degree of depolymerization and introducing carbonyl functionality into the resulting oligomers. Repolymerization of the oligomers readily produced dynamic polymer networks (DPNs) that are capable of closed-loop chemical recycling toward a biomass-based circular plastic economy.

## Results and Discussion

**Figure 2:**
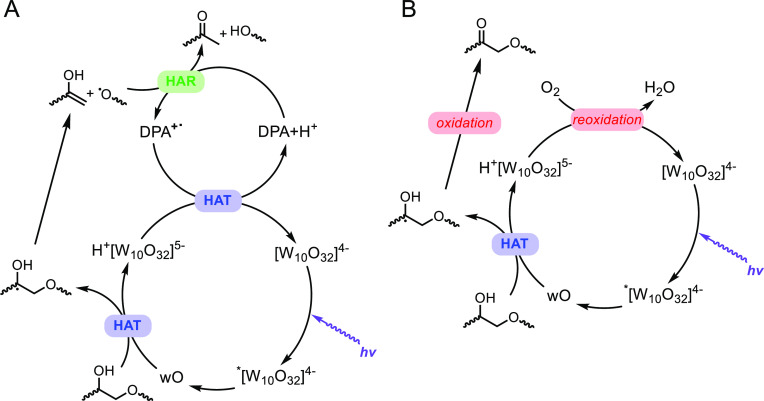
Two possible catalytic cycles of photocatalytic conversion of the β-O-4 motif with different cocatalysts. (a) Catalytic cycle of [W_10_O_32_]^4–^ in the presence of an electron mediator, DPA, which leads to the scission of the β-O-4 motif. (b) Catalytic cycle of [W_10_O_32_]^4–^ in the presence of an electron scavenger, O_2_, which leads to the oxidation of the β-O-4 motif.

Inspired by recent reports that show decatungstate ([W_10_O_32_]^4–^), a polyoxometalate anion, as an effective hydrogen-atom abstraction (HAA) reagent,^33−37^ we hypothesized that TBADT can be an efficient catalyst to directly target the abundant β-O-4 motifs in native lignin under mild conditions. Our proposed mechanism for the HAT mediated by TBADT involves hydrogen extraction from the substrate to result in an α-C radical upon irradiation, as shown in Figure 2. It can then lead to the scission of the β-C–O bond, producing an enol and an oxyl radical. The enol undergoes tautomerization to yield a ketone, and the oxyl radical receives the previously extracted hydrogen to produce an alcohol.^18−21^ It is noted that bond scission could also take place between the α-C–C bond, yielding an aldehyde and a methyl ether.^17^ In either case, the overall reaction involves a hydrogen-atom return (HAR). Previous reports have shown that the presence of an electron mediator, such as 9,10-diphenylanthracene (DPA), can lend its electron to the radical^38,39^ and facilitate HAR to promote bond scission (Figure 2a). In an alternative pathway, the α-C radical may be transformed into a stable carbonyl via oxidation, as shown in Figure 2b, in which case an electron scavenger would be necessary to turn over the catalyst by extracting the hydrogen.^37^

**Figure 3:**
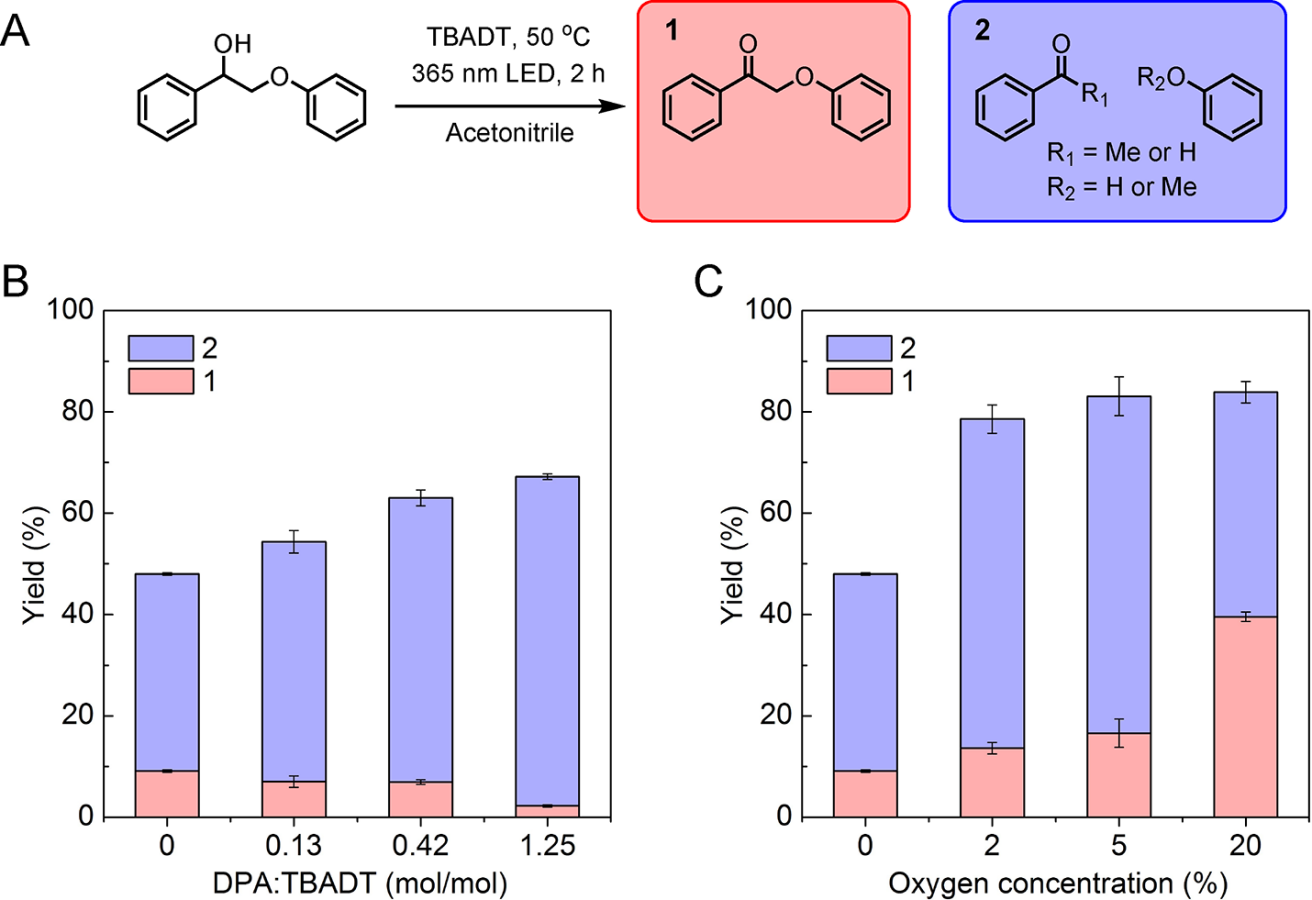
Photocatalytic conversion of the model compound, PPol, under varying conditions. (a) Two types of PPol reaction products in the presence of UV light: (1) oxidation and (2) bond scission. (b) Comparison of the yields and distribution of different products with varying DPA amount. (c) Comparison of the yields and distribution of different products with varying O_2_ concentration.

To test the proposed mechanism involving the β-O-4 motif, we next carried out photocatalytic reactions on a model compound, 2-phenoxy-1-phenylethanol (PPol), which has been used as a testing platform for the study of lignin chemistry by other reports.^18,19,21,40,41^ In a typical experiment, 50 μmol of PPol was mixed with 1.6 μmol of TBADT in 1 mL of acetonitrile, and the resulting solution was heated to 50 °C using a water bath under illuminated by a 200 W UV LED light centered at ca. 365 nm (see Supporting Information, SI, for more details). The reaction was performed under 1 bar of N_2_. As discussed above, two types of reactions are expected from this system, a redox neutral process that breaks down the β-C–O (or the α-C–C) bond or an oxidation reaction that preserves the β-O-4 motif but produces a carbonyl (Figure 3a). For a typical 2 h reaction, 4.56 μmol of 2-phenoxy-1-phenylethanone (PPone, compound **1** in Figure 3a) was detected, accounting for 9.1% oxidation of the starting material (PPol). For the same reaction, 19.4 μmol of benzaldehyde or acetophenone (compounds **2** in Figure 3a) or both was measured, reporting 38.9% bond scissions of the starting material. Also, 26.0 μmol of unreacted PPol was measured, which is consistent with the calculated conversion of 48.0% (Figure 3b). Adding DPA as an electron mediator under otherwise identical conditions promoted the selectivity toward bond scission. Of the converted starting material, 81.0% underwent bond breaking when no DPA was added; the selectivity increased to 96.7% when 125 mol % of DPA (relative to TBADT) was used (Figure 3b). Also increased was the total conversion, from 48.0% without DPA to 67.2% with 125 mol % of DPA. The increase in conversion was attributed to the improved TBADT turnover by DPA. As shown in Figure 2a, when DPA^+•^ extracts hydrogen from the reduced TBADT, it facilitates the restoration of TBADT to the initial state, ready for the next catalytic cycle. Taken as a whole, it is concluded from this series of experiments that TBADT is effective in cleaving PPol and introducing carbonyl functional groups, and addition of electron mediators can further promote this reaction.

We propose that the carbonyl (C=O) groups can serve as a convenient reactive handle to form a dynamic imine bond by reacting with amines.^42−46^ Therefore, it is of critical importance to control the density of C=O during the depolymerization of native lignin. Too few C=O will likely make it difficult to repolymerize the resulting oligomers; too many C=O would mean that the reaction pathway is shunted toward the oxidation pathway, causing incomplete depolymerization. A unique advantage offered by our photocatalytic depolymerization approach is the ability to control the reaction pathways by the introduction of exogenous electron scavengers. When the reaction proceeds in a redox-neutral fashion, it favors the depolymerization of native lignin; when it undergoes oxidation, it is more effective in increasing C=O densities. Such a selectivity is new and significant because it will allow us to fine tune the degree of depolymerization of native lignin while controlling the density of key functional groups for subsequent repolymerization. To test this understanding, the following set of experiments was carried out on the model molecule of PPol. As shown in Figure 3c, addition of O_2_ as an electron scavenger to the reaction system led to increased selectivity toward oxidation, changing the selectivity toward compound **1** from 19.0% with no O_2_ to 47.2% with 20% of O_2_ in N_2_ under otherwise identical reaction conditions. The corresponding stoichiometry of C=O increased from 2.24 mmol/g without O_2_ to 3.94 mmol/g with 20% of O_2_. It was observed that even without intentionally added electron scavengers, TBADT was capable of extracting electrons and protons from the substrate, acting as an effective oxidant, which helped explain why an appreciable amount of oxidation products (19.0%) was present under a pure N_2_ atmosphere. Our control experiments further proved that the concentration of oxidation products scaled with the amount of TBADT used (Figure S2). Replacing all N_2_ with 100% O_2_ led to overoxidation and diminished products of the desired compounds **1** or **2** (Figure S3). It was also observed that the addition of O_2_ significantly improved the conversion of PPol. For instance, only 2% of O_2_ increased the conversion from 48.0% to 78.6%. In the absence of electron mediators, the improved conversion is likely due to more TBADT turnovers, as shown in Figure 2b. These experiments carried out on the model compound, PPol, established that photocatalytic transformation of the β-O-4 motif can be achieved using TBADT following a HAT mechanism. It also showed that the reaction can be controlled between bond scission and oxidation. To further our understanding, we subjected another model system, 2-phenoxyl-1-phenylpropan-1,3-diol (PPdiol), to the same conditions and found no significant difference in conversion with similar product distributions (Table S2). With this knowledge in hand, we next turned our attention to the valorization of native lignin.

**Figure 4:**
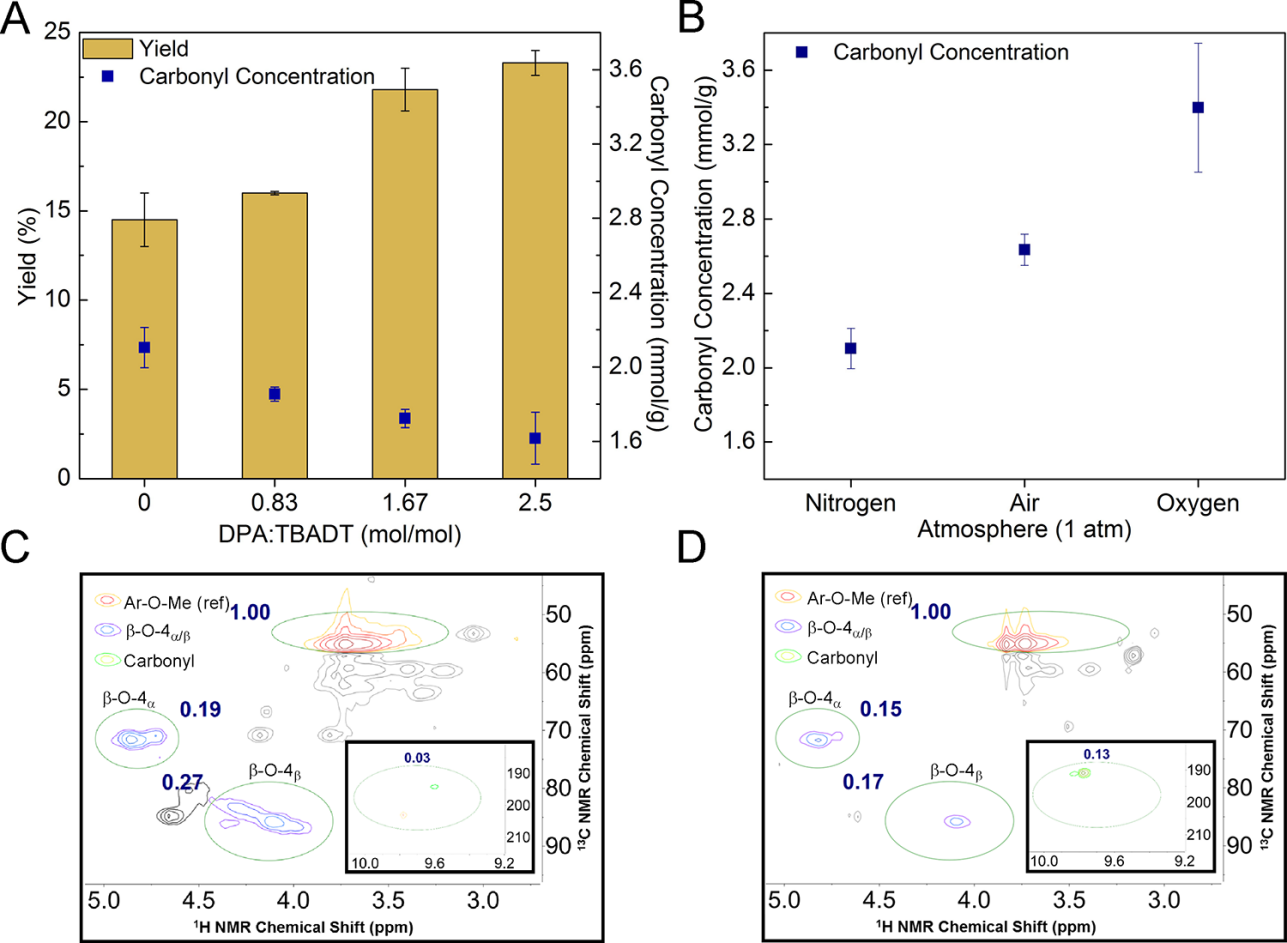
Photocatalytic conversion of native lignin to oligomers under varying conditions. (a) Comparison of the yield and C=O stoichiometry obtained from photocatalytic degradation of native lignin with varying DPA amount. (b) Comparison of the C=O stoichiometry obtained with varying atmosphere. (c) 2D-HSQC spectrum of organosolv lignin showing the integration of functional groups of interest. (d) 2D-HSQC spectrum of lignin oligomers showing the integration of functional groups of interest.

For this purpose, we conducted experiments on pretreated beech sawdust (*Fagus sylvatica*, Blegwood, catalog 874875223 on http://www.etsy.com) following reported procedures with minor modifications.^8,17^ A typical reaction was performed with a mixture of 2.50 g of woodmeal, 75 μmol of TBADT, and 30 mL of 2:1 acetone–acetonitrile mixture, which was then kept at 50 °C using a water bath with irradiation from the same UV LED light as noted above. The crude oligomers were purified with silica gel and neutral aluminum oxide column chromatography to remove the remaining catalysts (Figure S4). In characterizing the products, attention was focused on soluble oligomer products, and two key figures of merit were quantified, namely, the yield (the weight of oligomer divided by Klason lignin amount in the raw material;^8^ see SI for detailed calculations) and the C=O stoichiometry (Figure S5). With only TBADT present, a yield of 14.5% was obtained, and the products featured a C=O stoichiometry of 2.10 mmol/g. Adding DPA as an electron mediator increased the yield but decreased the C=O stoichiometry, as expected. For instance, with 250 mol % of DPA, the yield was up to 23.3% and the C=O stoichiometry was down to 1.62 mmol/g (Figure 4a). Conversely, when the reaction was carried out in different atmospheres, the presence of O_2_ as an electron scavenger resulted in significant increases of the C=O stoichiometry, from 2.10 mmol/g in pure N_2_ to 3.40 mmol/g in pure O_2_ (Figure 4b), with only modest changes to the yield (Figure S6). Importantly, the reaction could be performed in ambient air, and low concentrations of water or CO_2_ did not appear to influence the process.

To further confirm that β-O-4 bond cleavage took place, 2-dimensional heteronuclear single-quantum coherence spectroscopy (2D-HSQC) NMR (Figures S7 and S8) experiments were conducted using organosolv lignin^47^ as a soluble surrogate to native lignin (Figure 4c and 4d). The use of organosolv lignin as a soluble surrogate of native lignin is well documented in the literature.^17,22^ The 2D-HSQC NMR spectrum of organosolv lignin was consistent with the literature, showing the presence of β-O-4_α_- and β-O-4_β_/β′-O-4_β_-type bonds at ^1^H 4.6–5.0 ppm, ^13^C 66.3–76.5 ppm and ^1^H 3.8–4.5 ppm, ^13^C 78.9–92.5 ppm, respectively.^47−49^ Similar to other reports,^18,47^ the aryl methoxy (Ar–OMe) contours (^1^H 3.2–4.1 ppm, ^13^C 49.5–56.6 ppm) were used as an internal standard for comparison of the two samples as they remained unchanged by the photocatalytic degradation. A direct comparison was made by setting the integration of the Ar–OMe peak as 1.00 and integrating the same regions of interest in both samples. After the photocatalytic degradation of native lignin to oligomers, the integration of the contours of the β-O-4_α_ and β-O-4_β_/β′-O-4_β_ bonds showed a decrease from 0.19 to 0.15 and from 0.27 to 0.17, respectively, suggesting a partial cleavage of these bonds. In addition, the increase in intensity of the contours in the aldehyde region (^1^H 9.3–10.0 ppm, ^13^C 187.5–207.7 ppm) further supports the cleavage of the β-O-4 bond and the generation of the carbonyl functionalities in the resulting oligomers. The evidence strongly supports the proposed mechanisms as shown in Figure 1c and serves as a foundation to the photocatalytic lignin-first strategy. It is also noteworthy that the chemical integrity of the cellulose and hemicellulose was not affected by our photocatalytic conditions and can be recycled after the reaction (Figure S9).

**Figure 5:**
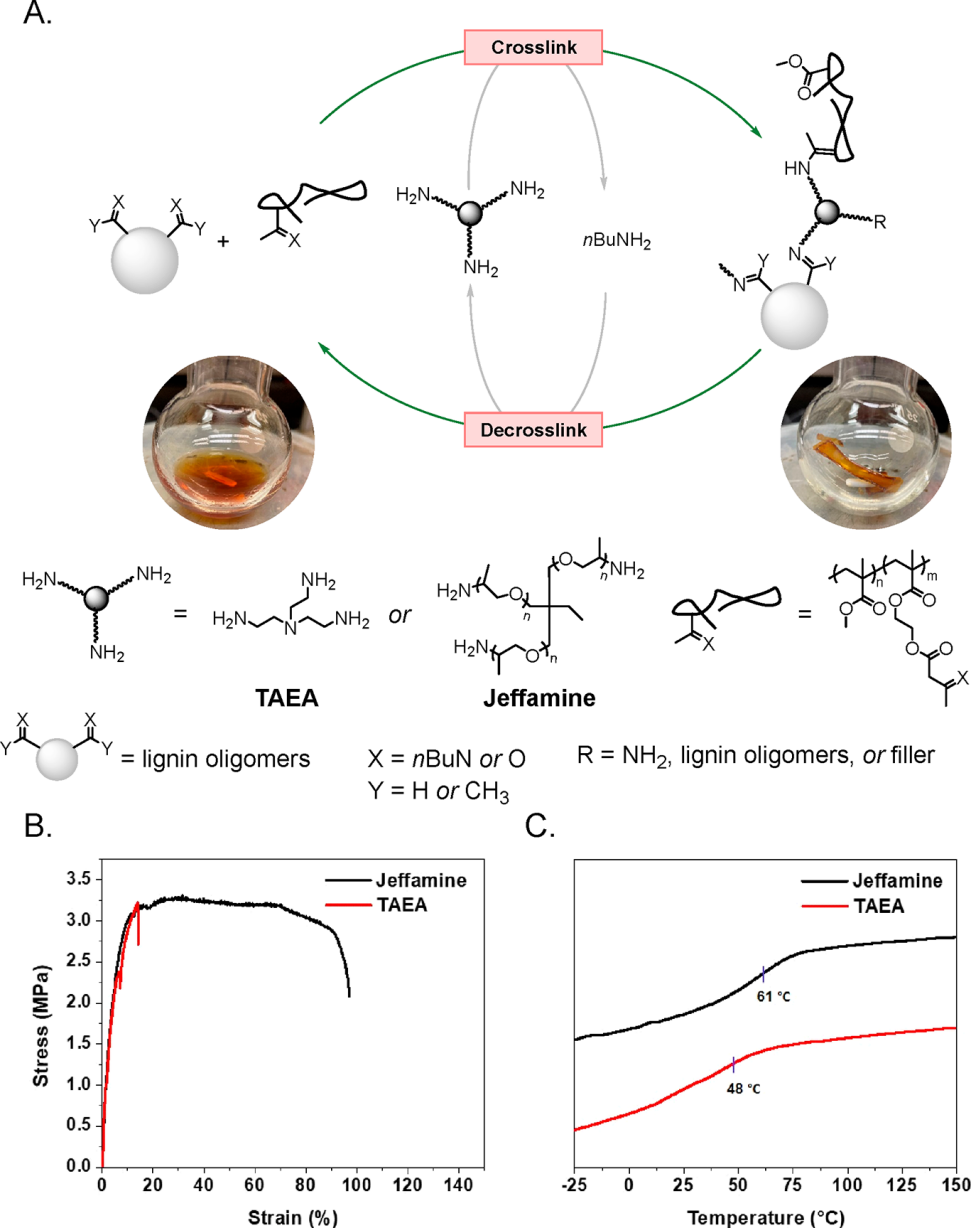
(a) Schematic illustration of the formation and recycling of lignin oligomer containing DPNs. (b) Tensile tests comparing different triamine cross-linked networks. (c) Glass transition temperatures of cross-linked networks as determined by DSC cooling curves.

With the ability to obtain lignin oligomers rich in carbonyl functional groups, we set out to create a lignin-based polymer network material by repolymerizing the lignin oligomers with triamine cross-linkers (Figure 5a). Inspired by the work of Sumerlin and co-workers,^50,51^ a copolymer of methyl methacrylate (MMA) and (2-acetoacetoxy)ethyl methacrylate (AAEMA) was used as a chemically recyclable filler to improve the mechanical strength of the network. The polymer network consisted of 40 wt % lignin oligomers and 60 wt % copolymer filler that was first cross-linked by a tris(2-aminoethyl)amine (TAEA) cross-linker. A substoichiometric amount (1 mol % amine groups with respect to total moles of cross-linkable carbonyls) of TAEA was found to be necessary to maintain the dynamic nature of the polymer network and avoid overhardening of the material. After curing at ambient temperature under vacuum for 24 h, tensile test revealed that the cross-linked network was brittle in nature, exhibiting a maximum stress of 3.25 MPa and maximum strain of 15%, likely due to the short nonflexible arms of TAEA (Figure 5b). To overcome this challenge, a commercially available trimethylolpropane tris[poly(propylene glycol), amine terminated] ether (Jeffamine) was used as the cross-linker. Jeffamine showed the ability to create a polymer network with improved tensile properties at the stoichiometric amount (100 mol % amine groups with respect to total moles of cross-linkable carbonyls). The maximum stress of the Jeffamine network was similar to that of the TAEA network at ca. 3.25 MPa, while the strain improved to ca. 98% before breaking. The Young’s modulus was similar for either the TAEA network or the Jeffamine network at 78 or 76 MPa, respectively. Thermal analysis of the two cross-linked networks by differential scanning calorimetry (DSC) revealed the glass transition temperature (*T*_g_) of the TAEA network to be 48 °C and the Jeffamine network to be 61 °C (Figure 5c). The higher *T*_g_ of the Jeffamine cross-linked network is likely a result of a higher cross-linking density given that the initial loading of cross-linker was higher. Notably, the polymer network was readily depolymerized into soluble lignin oligomers and the copolymer filler by treating the film with excess *n*-butylamine at 80 °C. Following extraction to remove excess *n*-butylamine, the residues were repolymerized by simply recuring at ambient temperature followed by drying under vacuum for 24 h. The recured sample exhibited similar mechanical properties as the original network (Figure S10). Overall, we demonstrated that the lignin oligomers generated from the catalytic depolymerization of native lignin can be repolymerized into mechanically robust polymer networks capable of closed-loop chemical recycling.

## Conclusions

We have developed a new approach to the direct conversion of native lignin to oligomers rich in carbonyl functionalities under photocatalytic conditions. The products were then used to prepare closed-loop, chemically recyclable DPNs. Toward this goal, TBADT as a photocatalyst made of earth-abundant elements was shown to be effective in either cleaving or oxidizing β-O-4 motifs through a HAT mechanism under conditions that would preserve cellulose and hemicellulose. The selectivity between decomposition and oxidation was controlled by adding exogenous electron mediators or electron scavengers. When applied to native lignin, the photocatalytic approach was shown to produce oligomers with up to 23.3% yield. The carbonyl stoichiometry was between 1.62 and 3.40 mmol/g, depending on the reaction conditions. 2D-HSQC results further supported that our photocatalytic system partially consumed β-O-4 motifs and generated high-value carbonyl functionalities. The produced oligomers could be repolymerized to form chemically recyclable polymer networks via imine bond formation. The DPNs were capable of closed-loop recycling. The present work not only represents a new strategy for lignin valorization under mild conditions beyond monomerization but also provides the ability to generate closed-loop recyclable lignin-based materials with promising material properties.

## Supporting Information Available

Supplementary results and methods including general experimental information and experimental details, 1H and 2D-HSQC NMR characterization, SEC traces, Klason lignin information, carbonyl quantification experiments, summary of PPol photocatalysis under varying conditions, summary of PPol vs PPdiol photocatalysis products, and summary of native lignin photocatalysis results under varying conditions (PDF)
