# Supplementary material for: Photocatalytic Depolymerization of Native Lignin toward Chemically Recyclable Polymer Networks
Source: ACS Cent Sci. 2022 Dec 28;9(1):48–55. doi: 10.1021/acscentsci.2c01257 (PMC9881207; doi:10.1021/acscentsci.2c01257)
Supplement: Supplementary file 1 — oc2c01257_si_001.pdf [file oc2c01257_si_001.pdf]

Supporting Information

Photocatalytic Depolymerization of Native Lignin Toward Chemically Recyclable Polymer Networks

Hongyan Wang,†,‡ Gavin J. Giardino,†,‡ Rong Chen,† Cangjie Yang,† Jia Niu,*,† Dunwei Wang*,†

† Department of Chemistry, Merkert Chemistry Center, Boston College, Chestnut Hill, Massachusetts 02467, United States.

‡ These authors contributed equally to this work.

email:

dwang@bc.edu

jia.niu@bc.edu

Table of Contents

I. Supplementary Figures

Figure S1	S3
Figure S2:	S3
Figure S3:	S4
Figure S4:	S4
Figure S5:	S6
Figure S6:	S7
Figure S7:	S7
Figure S8:	S8
Figure S9:	S8
Figure S10:	S9
Figure S11:	S11
Figure S12:	S17
Figure S13:	S17
Figure S14:	S18
Figure S15:	S18
**II. Supplementary Methods and Results**	
Klason Lignin Information	S5
Carbonyl Quantification Experiments	S5
Table S1 Summary of PPol photocatalysis under varying conditions	S13
Table S2 Summary of PPol vs. PPdiol photocatalysis products	S13
Table S3 Summary of native lignin photocatalysis results under varying conditions	S14
**III. References**	S19

I. Supplementary Figures

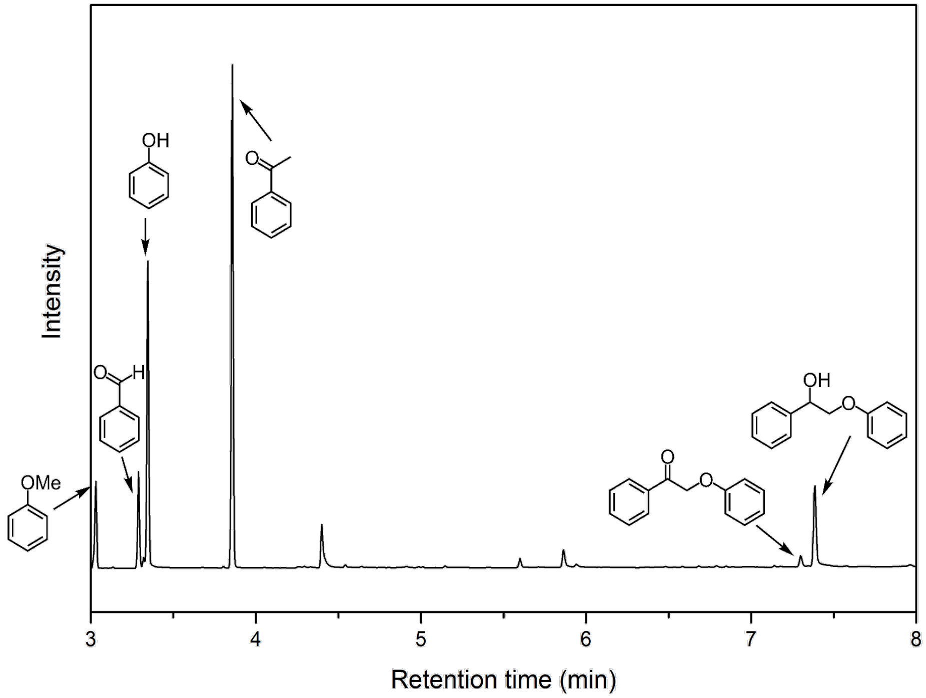

Figure S1: Gas chromatograph of the photocatalytic degradation of PPol.

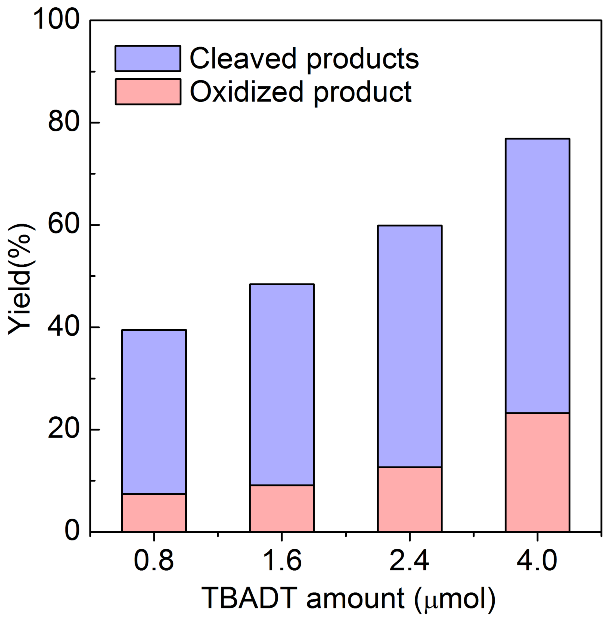

Figure S2: Comparison of the products of the photocatalysis of PPol using varying amounts of TBADT.

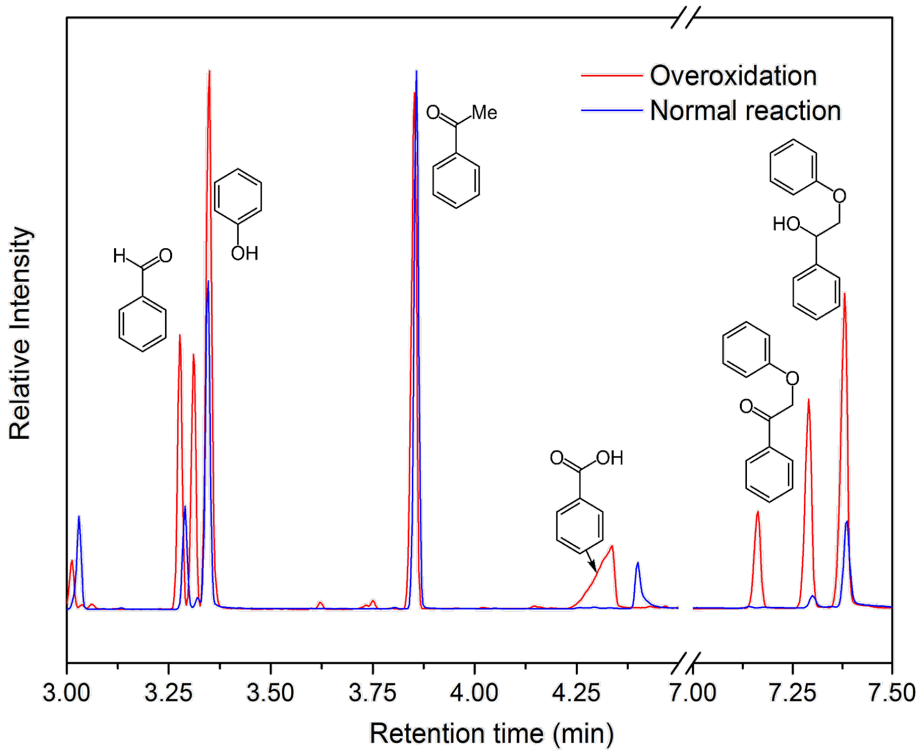

Figure S3: Gas chromatograph of the photocatalytic degradation of PPol under 100% oxygen atmosphere.

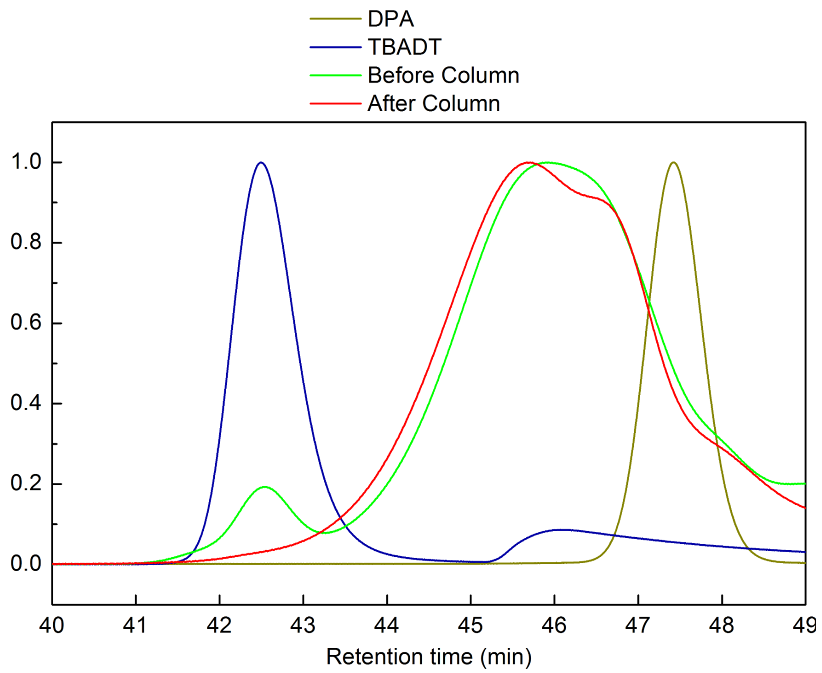

Figure S4: SEC of catalysts, crude oligomers, and purified oligomers.

Klason Lignin Information

Klason lignin content was measured according to a literature procedure in order to calculate the yield of lignin oligomers.[1] In a typical experiment, woodmeal (0.2 g) and 72% sulfuric acid (1.5 mL) were mixed in a glass vial, and stirred for 1 hour at 30 °C in a water bath. The mixture was diluted to 42 mL with DI water and transferred to an autoclave reactor. The solution was heated to 121 °C for 1 hour. After cooling to room temperature, the brown precipitate (Klason lignin) was separated by filtration. The solid was washed with DI water (3 x 20 mL). The Klason lignin was dried at 105 °C for 24 hours to afford the product (16.1 ± 0.1%).

$$Klason\ lignin\ content\ (\%) = \frac{Klason\ lignin\ product\ (g)}{Total\ biomass\ weight\ (g)} \times\ 100\%$$

Carbonyl Quantification Experiments

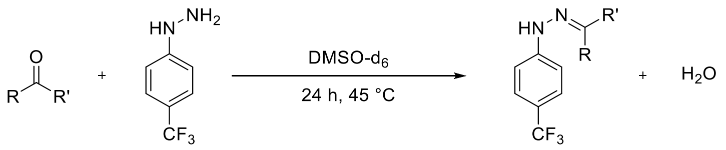

To calculate the amount of carbonyls in the lignin oligomers, a published method about using 4-(trifluoromethyl)phenylhydrazine to derivatize carbonyls to the corresponding hydrazone was applied.[2] For a typical quantification reaction, lignin oligomers (15 mg), 100 μL p-trifluoromethyl toluene (Internal standard, IS) in DMSO-d_6 solution (0.1682 μmol/mg, accurately weighed), 200 μL 4-(trifluoromethyl)phenylhydrazine in DMSO-d_6 solution (containing 70.5 mg 4-(trifluoromethyl)phenylhydrazine, 0.40 mmol), and 400 μL pure DMSO-d_6 were mixed in a 4 mL vial, and then homogenized in a vortex mixer. The mixture was then transferred to a NMR tube, and heated to 45 °C for 24 hours. Before NMR analysis,100 μL of relaxation agent solution in DMSO-d_6 (containing 2.8 mg chromium acetylacetonate) was added and homogenized. ^{19}F NMR was carried out with the following acquisition parameters: 90° pulse angle, relaxation delay = 3 sec, scans = 64, region = -50-70 ppm. IS was used as a reference peak set to -60.9 ppm, with the unreacted hydrazine peak at -59 ppm.

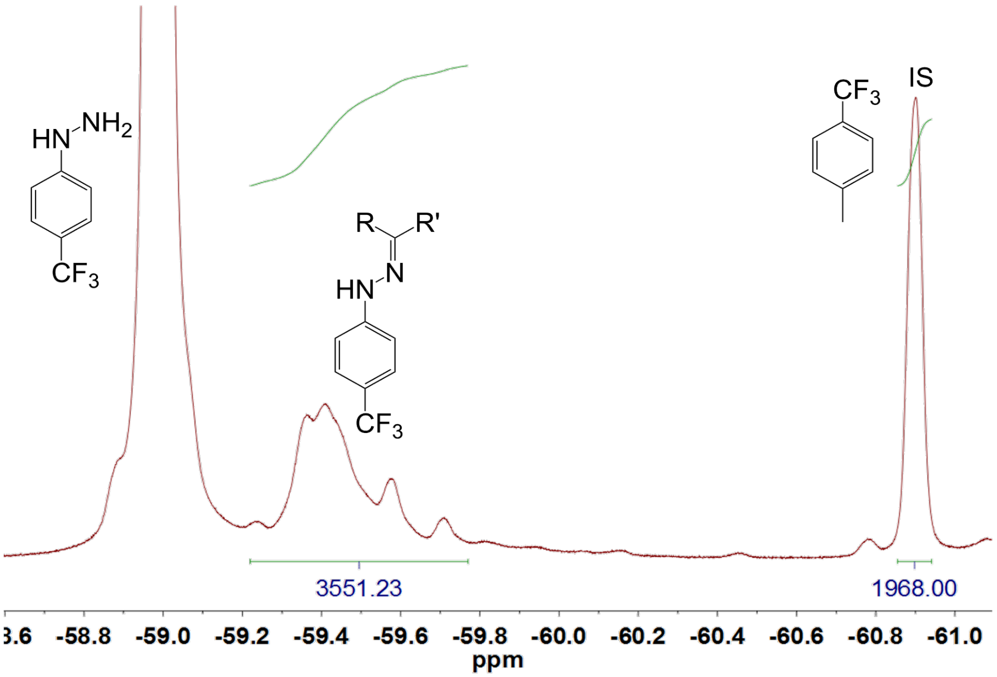

Figure S5: NMR of the hydrzaone formation for the quantification of carbonyl concentration in DMSO-d6 600 MHz.

The total amount of carbonyls present is calculated as follows:

Molarity of IS

$$117.0\,mg\ \times\ 0.1682\ \mu mol/mg = 19.68\ \mu mol\ Internal\ standard\ molecules$$

Concentration of carbonyls in sample

$$35.51\ \ \mu mol\ /\ 13.2\,mg = 2.69\ mmol/g$$

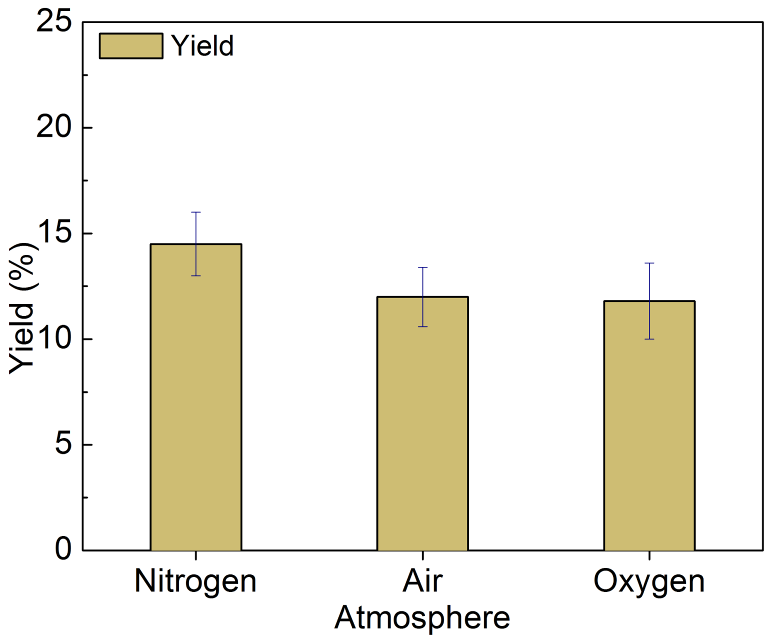

Figure S6: Comparison of the yields of the photocatalysis of native lignin to lignin oligomers under varying atmospheres.

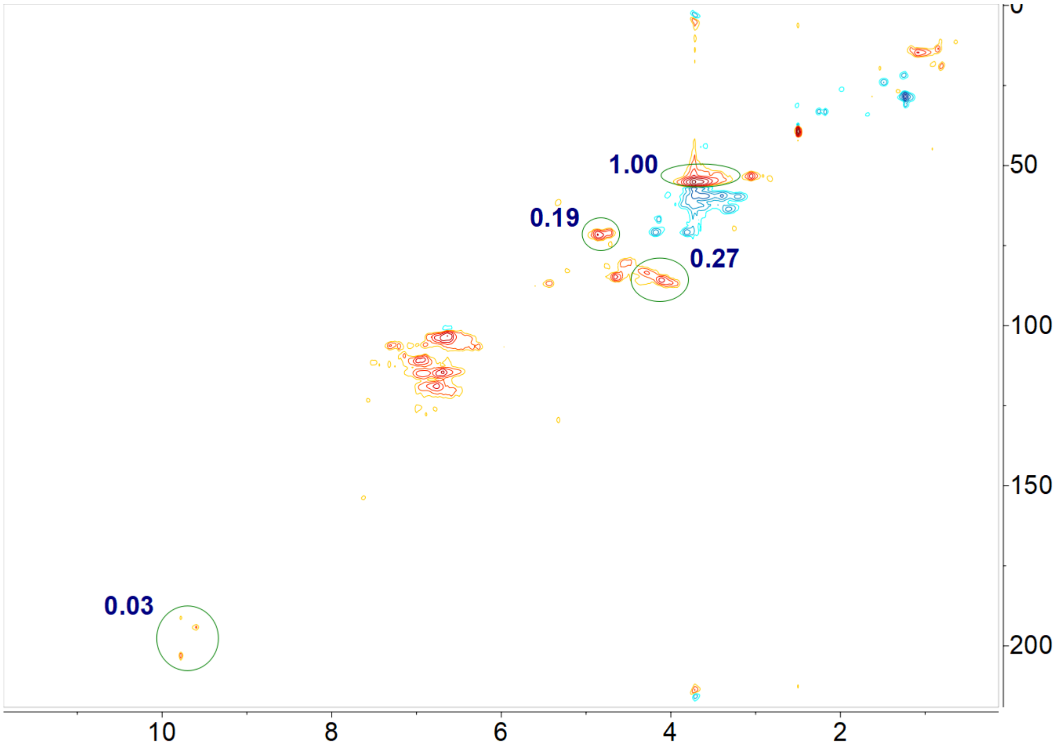

Figure S7: Full 2D-HSQC of organosolv lignin in DMSO-d_6, 500 MHz.

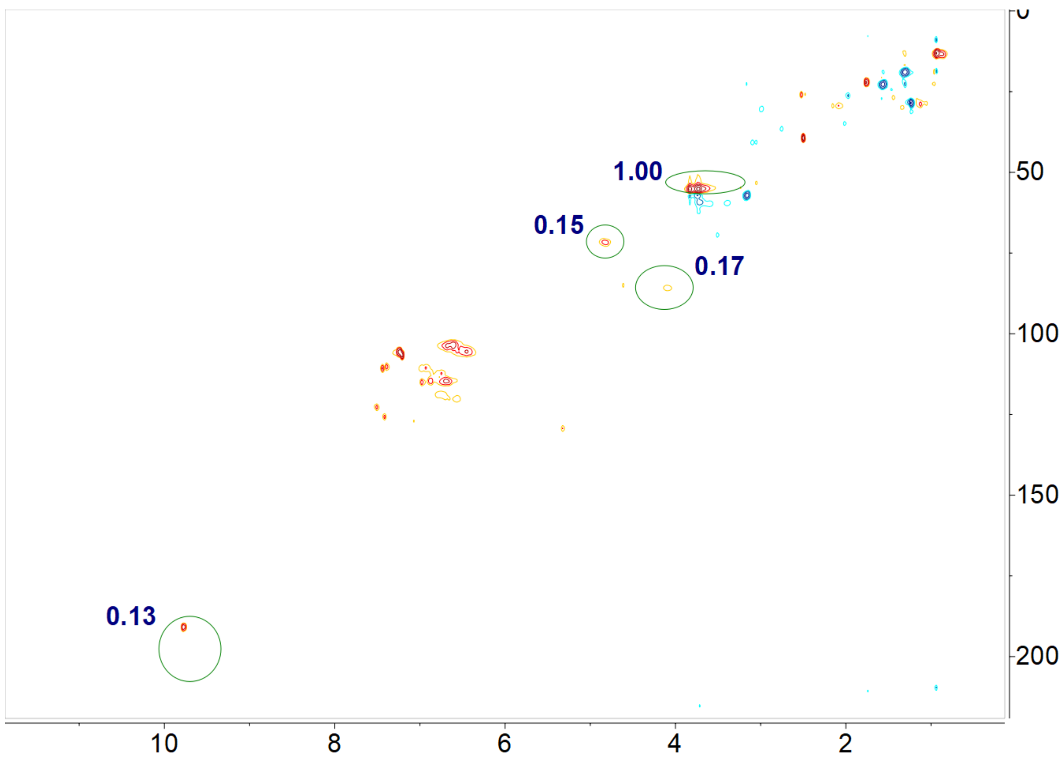

Figure S8: Full 2D-HSQC of lignin oligomers in DMSO-d6, 500 MHz.

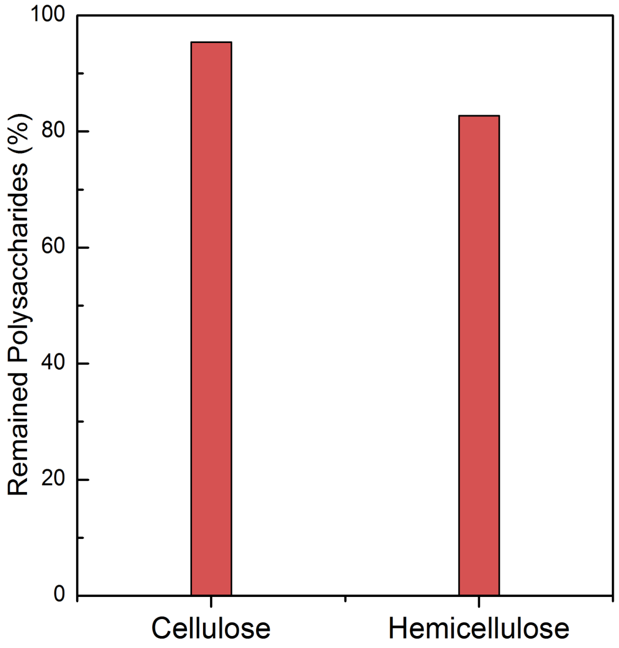

Figure S9: Quantification of remaining cellulose and hemicellulose after photocatalysis.

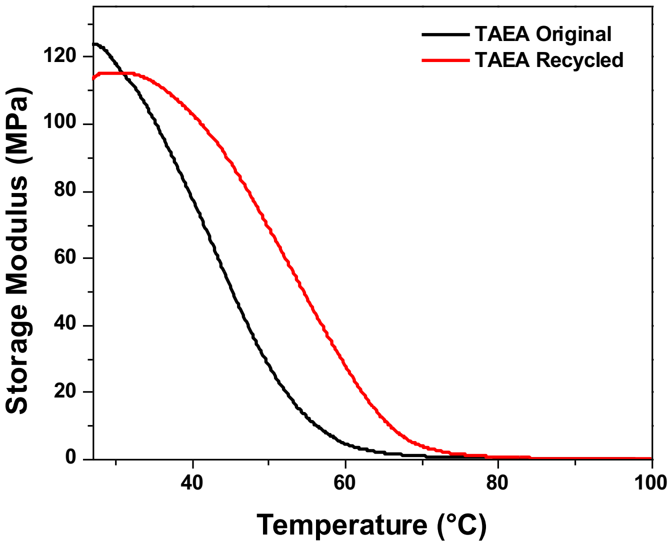

Figure S10: DMA analysis of original crosslinked TAEA network and recycled TAEA network.

II. Supplementary Methods and Results

Safety Statement

No unexpected or unusually high safety hazards were encountered.

Materials

All reactions were carried out under a nitrogen atmosphere unless otherwise stated. Nitrogen and oxygen gasses were purchased from airgas. Beech wood sawdust was purchased from Blegwood. All solvents including ethanol, acetone (AC), anhydrous acetonitrile (AN), dichloromethane (DCM), dioxane, ethyl acetate (EA), hexane (Hex), methanol (MeOH), and tetrahydrofuran (THF) were purchased from commercial sources and used without further purification unless otherwise stated. Dioxane was further dried with a drying column of a solvent system from Pure Process Technologies. NMR solvents including dimethyl sulfoxide (DMSO-d_6) and chloroform ($CDCl_3$) were purchased from Cambridge Isotope Laboratories or ACROS organics. Sodium tungstate dihydrate and tetrabutylammonium bromide (TBAB) were purchased from Acros organics. Diphenyl acthracene (DPA) was purchased from Alfa Aesar. 2-phenoxy-1-phenylethanol (PPol) and 4-Cyano-4-(dodecylsulfanylthiocarbonyl)sulfanylpentanoic acid were purchased from Ambeed. Methyl 2-methylprop-2-enoate (MMA) and *n*-butylamine were purchased from TCI. (2-acetoacetoxy)ethyl methacrylate (AAEMA), azobisisobutyronitrile (AIBN) (recrystallized from methanol prior to use), cellulose, hemicellulose, 4-(trifluoromethyl)phenylhydrazine, trimethylolpropane tris[poly(propylene glycol), amine terminated] ether (Jeffamine), and tris(2-aminoethyl)amine (TAEA) were purchased from Sigma Aldrich. MMA and AAEMA were passed through a basic aluminum oxide plug prior to use. SiliaFlash (P60 40-63 µM (230-400 mesh)) silica gel was purchased from SiliCycle and used for column chromatography. Basic aluminum oxide (Brockmann I 50-200 µm, 60A) was used for reagent purification. Neutral aluminum oxide (150 mesh) was used for oligomer purification. Thin Layer Chromatography (TLC) Silica Gel 60 F_{254}: Glass Plates 20 x 20 cm were used for TLC analysis. The polytetrafluoroethylene (PTFE) mold used for creating tensile test and DMA samples had the following measurements, respectively:

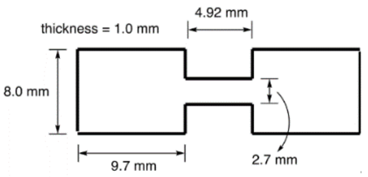

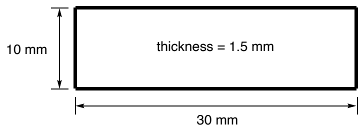

Characterization

All ^{1}H, ^{13}C, and ^{19}F NMR spectra were obtained using a Varian 600 MHz, Varian 500 MHz, Bruker 500 MHz equipped with a X nuclei optimized 5 mm double resonance BBO H&F CryoProbe Prodigy, or Bruker 500 MHz equipped with a CryoProbe (He) BBFO 5mm BBF/1H X nuclei optimized 5 mm double resonance BBFO, NMR spectrometer. Size exclusion chromatography (SEC) was carried out on a Tosoh's high-performance SEC system HLC-8320GPC containing TSKgel Alpha-M columns at 50 °C and a flow rate of 0.6 mL/min. The eluent was HPLC grade DMF with 0.01 M LiBr. Polystyrene standards (ReadyCal Kit, Sigma-Aldrich #81434) were used to determine the molecular weight and molecular weight distribution of the polymers. Prior to injection into the SEC, polymers were dissolved in DMF and filtered through a 0.20 µm PTFE filter. Differential scanning calorimetry (DSC) was performed using Tzero hermetic pans and lids on a TA Instrument Discovery DSC 250. Scans were performed from -50-200 °C over three cycles at a rate of 10 °C/min. An isotherm step for 5 minutes was used at both -50 and 200 °C. Tensile test was performed on an Instron 5543 universal testing system that was equipped with a 100 N load cell. The strain rate was set at 2 strain/min. Dynamic mechanical analysis (DMA) was carried out on a TA Instruments Discovery Dynamic Mechanical Analyzer 850.

Light Reactor Specifications and Setup

The light source was a 200 W UV LED light centered at 365 nm, from Howsuper, with the source number H6015-S-6868-LG-365nm.

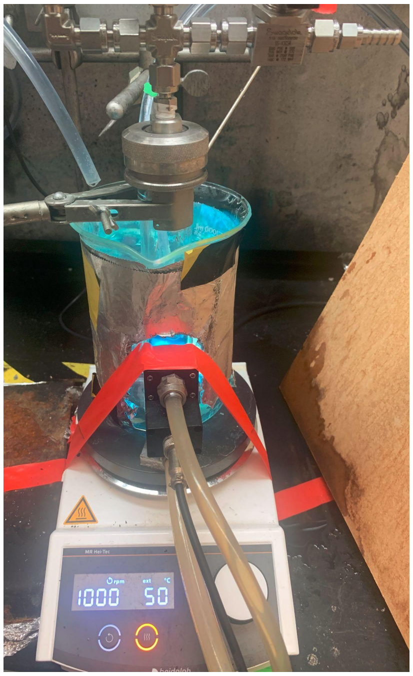

Figure S11: Light setup used for photocatalysis.

TBADT Catalyst Synthesis

$$4\ NBu_4^{+} + 10\ WO_4^{2-} + 16\ H^{+} \xrightarrow{90\ °C} (NBu_4)_4[W_{10}O_{32}] + 8\ H_2O$$

TBADT catalyst was synthesized according to a literature procedure with minor modifications.[3] TBAB (4.8 g, 15 mmol) and $Na_2WO_4 \cdot 2H_2O$ (10.0 g, 30 mmol) were dissolved in separate deionized (DI) water (50 mL and 100 mL, respectively) solutions. The solutions were acidified to pH 2 with concentrated hydrochloric acid. After heating to 90 °C, the solutions were mixed together. Precipitation was observed immediately, indicating the formation of TBADT. The slurry was stirred for 30 minutes in a 90 °C water bath, cooled to room temperature, and filtered with a Büchner funnel. The solid phase was washed with DI water and THF (3 x 30 mL), and dried in a vacuum oven at 90 °C overnight. The crude TBADT was further purified by recrystallization. The crude TBADT was refluxed in DCM (1 g: 20 mL) for 2 hours. The mixture was cooled on an ice bath, and then filtered to obtain pure TBADT as a transparent crystal with a light yellow color (4.02 g, 40.0% based on W).

Photocatalysis of PPol or PPdiol without DPA

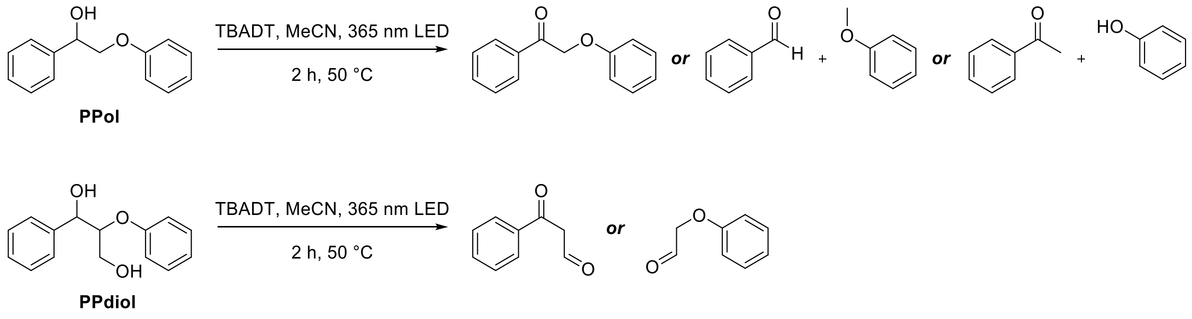

For a typical photocatalytic reaction of PPol or PPdiol without DPA, PPol (10.7 mg, 50 µmol) or PPdiol (12.2 mg, 50 µmol), TBADT (5.3 mg, 1.6 µmol), and AN (1 mL) were added to a 10 mL Schlenk tube. The solution was degassed by the freeze-pump-thaw method (3 x) such that the atmosphere of the schlenk tube was 1 atm nitrogen. The solution was then heated to 50 °C in a water bath and irradiated with UV light for 2 hours. The products were then analyzed and quantified by ^{1}H NMR (48.0% conversion, 9.1% PPone, 29.2% acetophenone, 9.7% benzaldehyde). See Table S1 for yield details.

Photocatalysis of PPol with DPA

The procedure is the same as the photocatalysis of PPol without DPA. DPA amounts used were as follows:

66.1 µg, 0.2 µmol

or

220 µg, 0.67 µmol

or

661 µg, 2.0 µmol

See Table S1 for yield details.

Photocatalysis of PPol with Oxygen:Nitrogen Mixture Atmosphere

Varying oxygen mixtures were prepared by mixing pure oxygen and pure nitrogen in a high-pressure reactor. For 2%, 5% and 20% oxygen, the partial pressures for oxygen and nitrogen were 0.5 bar/24.5bar, 0.5 bar/9.5bar, 2 bar/8 bar, respectively. The procedure is the same as the photocatalysis of PPol reaction without DPA; however, the oxygen:nitrogen gas mixtures were introduced instead of pure nitrogen during freeze-pump-thaw cycles. Oxygen percentages used are as follows:

2% oxygen

or

5% oxygen

or

20% oxygen

See Table S1 for yield details.

Table S1: Summary of PPol photocatalysis under varying conditions.

DPA (µmol)	Oxygen Concentration (%)	Conversion (%)	PPone (NMR Yield %)	Acetophenone (NMR Yield %)	Benzaldehyde (NMR Yield %)
0	0	48.0 ± 0.3	9.1 ± 0.3	29.2 ± 0.2	9.7 ± 0.3
0.2	0	54.4 ± 3.3	7.0 ± 1.1	38.4 ± 1.1	9.0 ± 1.1
0.67	0	63.0 ± 2.0	6.9 ± 0.5	46.8 ± 1.4	9.3 ± 0.2
2.0	0	67.2 ± 0.3	2.2 ± 0.2	55.8 ± 0.1	9.2 ± 0.7
0	2	78.6 ± 3.7	13.6 ± 1.1	39.4 ± 1.8	25.6 ± 1.6
0	5	83.1 ± 1.0	16.6 ± 2.8	41.6 ± 0.9	24.9 ± 2.9
0	20	83.9 ± 1.2	39.6 ± 0.9	30.8 ± 2.4	13.5 ± 0.3

Table S2: Summary of PPol vs. PPdiol photocatalysis products.

Substrate	DPA (µmol)	O_2 (%)	Conversion (%)	PPone (NMR Yield %)	Acetophenone (NMR Yield %)	Benzaldehyde (NMR Yield %)	Γ-Aldehyde (NMR Yield %)
PPol	0	0	48.0 ± 0.3	9.1 ± 0.3	29.2 ± 0.2	9.7 ± 0.3	0
PPdiol	0	0	54.8 ± 3.5	30.0 ± 0.6	16.8 ± 3.0	8.0 ± 0.6	1.5 ± 0.1

Photocatalysis of Native Lignin without DPA

For a typical photocatalytic reaction of native lignin, woodmeal (2.50 g), TBADT (250 mg, 75 µmol), and AC:AN (30 mL 2:1) were mixed together in a 3 oz pressure reaction vessel. The system was degassed by freeze-pump-thaw in the same manner as the photocatalysis of PPol. The reactor was heated in a water bath to 50 °C and stirred for 48 hours under UV light irradiation. The oligomer solution was then collected by filtering the resulting slurry. The solution was concentrated under reduced pressure and the residue was resuspended in THF and sonicated for 5 minutes. The solution was then passed through a syringe filter (PET-45/25 polyester membrane with pore diameter of 0.45 µm, and membrane diameter of 25 mm) to remove the precipitated TBADT. The crude oligomers were purified with silica gel column chromatography. A mixture of hexane and ethyl acetate (8:2) was used to remove non-polar impurities. The gradient was then switched to DCM and methanol (1:0-0:1) to obtain the remaining oligomers. The collected oligomers were then subjected to a neutral aluminum oxide plug (DCM:MeOH 1:0-0:1) to further purify the oligomers (58.2 mg, 14.5%). See Table S2 for yield details.

Photocatalysis of Native Lignin with DPA

The procedure is the same as the photocatalysis of native lignin without DPA. DPA amounts used were as follows:

20.7 mg, 62.5 µmol

or

41.3 mg, 125 µmol

or

62.0 mg, 187.5 µmol

See Table S2 for yield details.

Photocatalysis of Native Lignin with Different Atmospheres

The procedure is the same as the photocatalysis of native lignin without DPA; however, either oxygen gas or air (1 atm) were introduced instead of pure nitrogen during freeze-pump-thaw cycles.

See Table S2 for yield details.

DPA (µmol)	Atmosphere	Oligomers (Isolated Yield %)	Carbonyl Concentration (mmol/g)
0	Nitrogen	14.5 ± 1.5	2.10 ± 0.11
62.5	Nitrogen	16.0 ± 0.1	1.85 ± 0.04
125	Nitrogen	21.8 ± 1.2	1.72 ± 0.05
187.5	Nitrogen	23.3 ± 0.7	1.62 ± 0.14
0	Air	12.0 ± 1.4	2.64 ± 0.08
0	Oxygen	11.8 ± 1.8	3.40 ± 0.35

Table S3: Summary of native lignin photocatalysis results under varying conditions.

Preparation of Organosolv Lignin

Organosolv lignin for 2D-HSQC NMR was synthesized according to the literature.[4] Pretreated woodmeal (5 g), 80% ethanol aqueous solution (40 mL), and concentrated hydrochloric acid (0.8 mL) were added to a 100 mL round-bottom flask. The mixture was refluxed at 80 °C for 5 hours at which point the mixture was filtered. The residue was washed with 100% ethanol (4 x 5 mL). The filtrate was collected and concentrated under reduced pressure. The remaining solid from the filtrate was re-dissolved in acetone (5 mL). The solution was then precipitated into DI water by dropwise addition (100 mL). Upon addition to the water, a brown-pink precipitate formed. The solution was filtered and the collected solid was dried in a vacuum oven at 45 °C overnight to afford the product (0.25 g, 5%).

2D-HSQC NMR Experimental Details

To obtain 2D-HSQC spectra of organosolv lignin and the generated lignin oligomers, 25 mg of each sample was dissolved in 600 µL of DMSO-d_6. The spectra were run on a Bruker 500 MHz equipped with a CryoProbe (He) BBFO 5mm BBF/1H X nuclei optimized 5 mm double resonance BBFO. The pulse sequence selected was hsqcedetgpsisp2.3. The acquisition parameters were as follows: NS = 8, AQ = 0.08704 sec, D1 = 1 sec, 1SW = 220 ppm, O1P = 6.000, 2SW = 11.7592, O2P = 110.00, DB = 0, 1TD = 256, 2TD = 1024. The data was processed using MestReNova x64-14.1.1-24571. DMSO-d_6 was used as a reference peak set to ^{1}H 2.50 ppm, ^{13}C 39.52 ppm. The raw data was auto phase corrected and auto baseline corrected before integration. The contours were integrated using the following regions: Ar-OMe (^{1}H 3.2-4.1 ppm, ^{13}C 49.5-56.6 ppm) (internal standard), β-O-4_{α} (^{1}H 4.6-5.0 ppm, ^{13}C 66.3-76.5 ppm), β-O-4_{β}/β'-O-4_{β} (^{1}H 3.8-4.5 ppm, ^{13}C 78.9-92.5 ppm), aldehyde (^{1}H 9.3-10.0 ppm, ^{13}C 187.5-207.7 ppm).

Wood Sawdust (Native Lignin) Pretreatment

Beech wood sawdust was pretreated according to a literature procedure with minor modifications.[5] The wood sawdust (1 g:10 mL) was suspended in an ethanol-water solution (4:1) and sonicated for 2 hours to remove most of the extractives. The solution was filtered, and the solid was washed with acetone (3 x 100 mL), and dried in a vacuum oven at 45 °C for a minimum of 48 hours. The solid was then oven-dried in 2 hour intervals at 105 °C until the weight change was <5% indicating a <5% moisture content. The dry sawdust was then knife-milled through a 1 mm screen. The wood powder was sealed and stored at 0 °C.

Control Experiments with Cellulose and Hemicellulose

The photocatalysis on cellulose and hemicellulose featured the same conditions and additives as typical woodmeal photocatalysis above, with the only difference being the reactant: using pure cellulose or hemicellulose (1.00 g) instead of woodmeal (2.50 g). After the reaction, the solution was filtered and washed with AN (3 x 5 mL), to remove TBADT, dried in vacuum oven overnight, and weighed to calculate the remaining percentage (weight after reaction ÷ weight before reaction) of each species. After photocatalysis, cellulose (95.4%) and hemicellulose (82.7%) were recovered. See Figure S9.

Synthesis of Polymer Filler

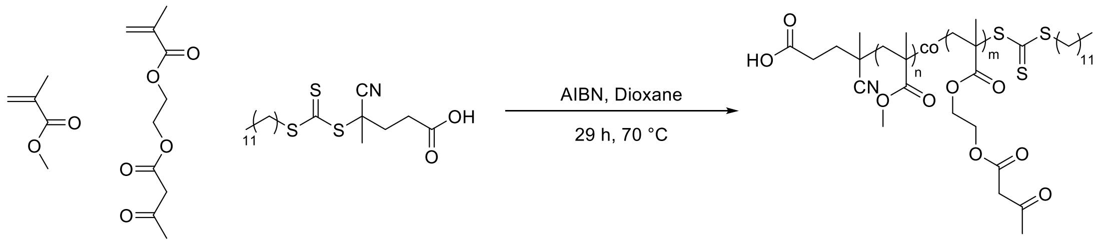

MMA (2.00 mL, 18.76 mmol) and AAEMA (358.14 µL, 1.88 mmol) were added to a flask and dissolved in dioxane (15 mL). AIBN (4.62 mg, 28.14 µmol) was added as a dioxane (1 mL) solution. The system was sealed with a septum and subjected to 3 rounds of freeze-pump-thaw to remove oxygen. The solution was heated to 70 °C for 29 hours at which point the solution was concentrated by half and precipitated into hexane (3 x 45 mL). The product was obtained as a yellow solid (2.00 g). The NMR spectrum is consistent with the literature[6] and is shown below (Figure S12) for the calculation of AAEMA incorporation.

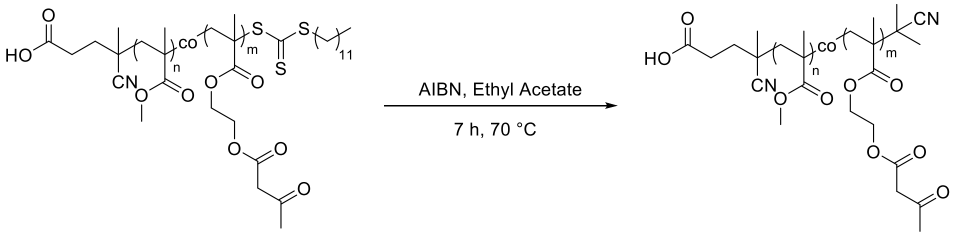

4-cyano-8-dodecylsulfanylcarbothioylsulfanyl-6-methoxycarbonyl-4,6,8-trimethyl-9-oxo-10-[2-(3-oxobutanoyloxy)ethoxy]decanoic acid (2.00 g, 2.73 mmol) and AIBN (30.81 mg, 187.6 µmol) were dissolved in EA (27 mL). The system was degassed with nitrogen for 25 minutes at which point the system was sealed with a septum and heated to 70 °C for 7 hours. The solution was concentrated by half and precipitated into hexane (3 x 45 mL). The product was obtained as a white solid (1.85 g). The NMR spectrum is consistent with the literature[6] and is shown below (Figure S13) for the calculation of AAEMA incorporation.

TAEA Crosslinking Procedure

As an example, lignin (34 mg, f = 2.892 mmol/g), and copolymer (52 mg, M_n = 17.6 kDA) were dissolved in THF (0.296 mL) in a 1.5 mL vial. From a stock solution of TAEA (10 µL in 990 µL of THF) was transferred (6.78 µL, 4.53 x 10^{-4} mmol) to the lignin solution. The solution was mixed in a circular motion and transferred within 10 seconds to a PTFE mold via pipette, in order to avoid cross-linking in the mixing vessel. Any air bubbles were carefully removed with the tip of a needle. The solution was allowed to sit at ambient conditions and undergo solvent evaporation for 18 hours at which point the film was carefully removed from the mold. The sample was cured under vacuum at room temperature for 24 hours to remove residual solvents.

Jeffamine Crosslinking Procedure

As an example, lignin (34 mg, f = 2.800 mmol/g), and copolymer (52 mg, M_n = 17.6 kDA) were dissolved in THF (0.250 mL) in a 1.5 mL vial. From a stock solution of Jeffamine (200 µL in 300 µL of THF) was transferred (44.89 µL, 4.0 x 10^{-2} mmol) to the lignin solution. The solution was mixed in a circular motion and transferred within 10 seconds to a PTFE mold via pipette, in order to avoid cross-linking in the mixing vessel. Any air bubbles were carefully removed with the tip of a needle. The solution was allowed to sit at ambient conditions and undergo solvent evaporation for 18 hours at which point the film was carefully removed from the mold. The sample was cured under vacuum at room temperature for 24 hours to remove residual solvents.

TAEA Recycling Procedure

The obtained film was suspended in *n*-butylamine (4 mL) and heated to 80 °C until all solid was dissolved (4-18 hours). The solution was concentrated under reduced pressure. The residue was then subjected to the initial crosslinking conditions.

Jeffamine Recycling Procedure

The obtained film was suspended in *n*-butylamine (4 mL) and heated to 80 °C until all solid was dissolved (4-18 hours). The solution was partially concentrated to remove about half of the *n*-butylamine. DCM and water (20 mL) were added. The aqueous phase was extracted with DCM (3 x 20 mL). The combined organic phases were washed with water (5 x 100 mL). The combined organic phases were dried over anhydrous sodium sulfate, filtered, and concentrated under reduced pressure. The residue was then subjected to the initial crosslinking conditions.

Characterization (NMR and SEC)

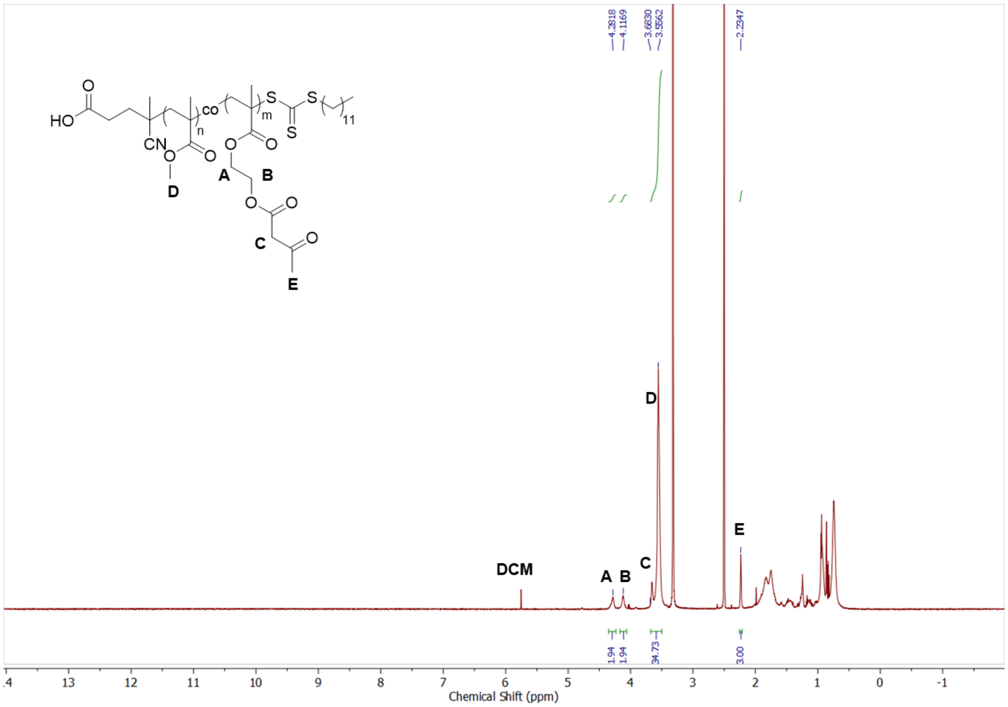

Figure S12: ^{1}H NMR of copolymer filler before end group exchange in DMSO-d_6, 600 MHz.

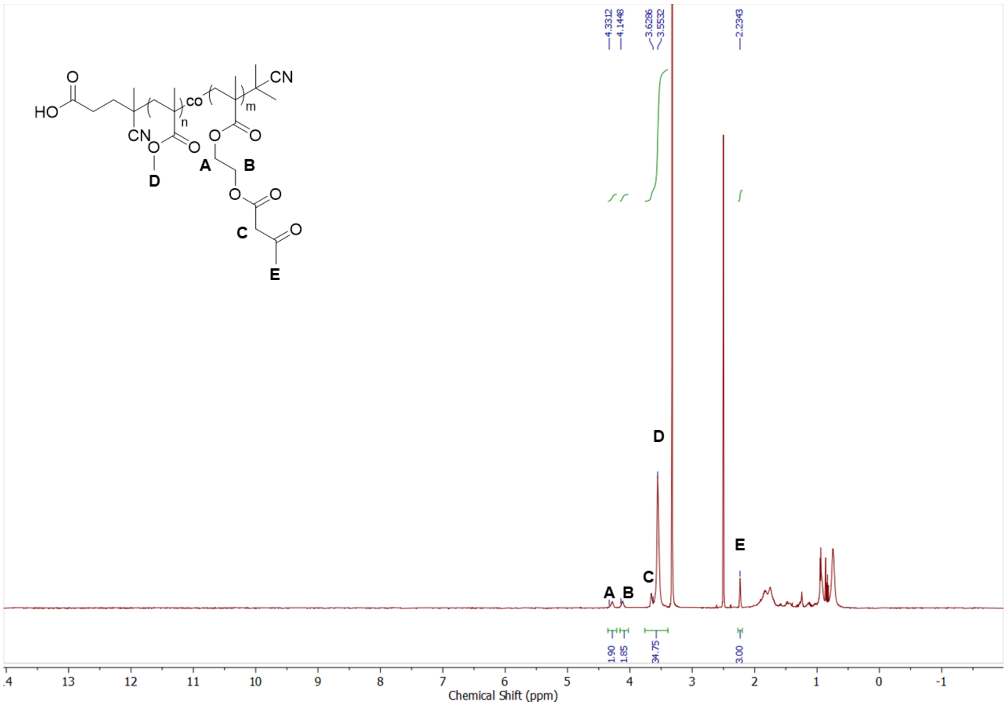

Figure S13: ^{1}H NMR of copolymer filler after end group exchange in DMSO-d_6, 600 MHz.

AAEMA Incorporation Calculation

To calculate the percentage of AAEMA incorporation, the equation from Sumerlin and coworkers was used.[6]

$$I = integration\ of\ peak$$

$$\%\ AAEMA = \frac{I_e}{(I_{c+d}) - 2 + 3} \times 100\%$$

$$\%\ AAEMA = \frac{3}{34.8 - 2 + 3} \times 100\% = 8.4\%\ incorporation\ before\ and\ after\ chain\ end\ exchange$$

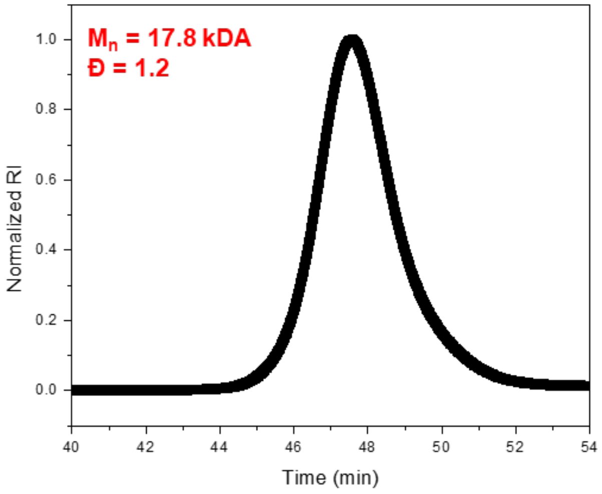

Figure S14: SEC trace of copolymer filler before end group exchange.

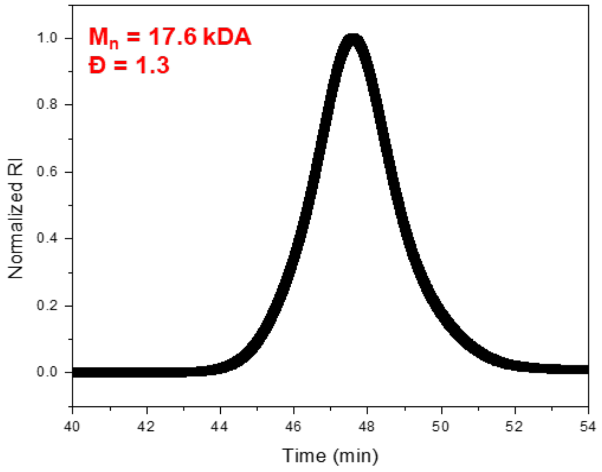

Figure S15: SEC trace of copolymer filler after end group exchange.

III. References

1. Abu-Omar, M. M.; Barta, K.; Beckham, G. T.; Luterbacher, J. S.; Ralph, J.; Rinaldi, R.; Román-Leshkov, Y.; Samec, J. S.; Sels, B. F.; Wang, F., Guidelines for performing lignin-first biorefining. *Energy & Environmental Science* **2021,** *14* (1), 262-292.
2. Constant, S.; Lancefield, C. S.; Weckhuysen, B. M.; Bruijnincx, P. C., Quantification and classification of carbonyls in industrial humins and lignins by 19F NMR. *ACS Sustainable Chemistry & Engineering* **2017,** *5* (1), 965-972.
3. Protti, S.; Ravelli, D.; Fagnoni, M.; Albini, A., Solar light-driven photocatalyzed alkylations. Chemistry on the window ledge. *Chemical communications* **2009,** (47), 7351-7353.
4. Zijlstra, D. S.; de Santi, A.; Oldenburger, B.; de Vries, J.; Barta, K.; Deuss, P. J., Extraction of lignin with high β-O-4 content by mild ethanol extraction and its effect on the depolymerization yield. *JoVE (Journal of Visualized Experiments)* **2019,** (143), e58575.
5. Wu, X.; Fan, X.; Xie, S.; Lin, J.; Cheng, J.; Zhang, Q.; Chen, L.; Wang, Y., Solar energy-driven lignin-first approach to full utilization of lignocellulosic biomass under mild conditions. *Nature catalysis* **2018,** *1* (10), 772-780.
6. Lessard, J. J.; Garcia, L. F.; Easterling, C. P.; Sims, M. B.; Bentz, K. C.; Arencibia, S.; Savin, D. A.; Sumerlin, B. S., Catalyst-Free Vitrimers from Vinyl Polymers. *Macromolecules* **2019,** *52* (5), 2105-2111.
